# Caudal Edge of the Liver in the Right Upper Quadrant (RUQ) View Is the Most Sensitive Area for Free Fluid on the FAST Exam

**DOI:** 10.5811/westjem.2016.11.30435

**Published:** 2017-01-19

**Authors:** Viveta Lobo, Michelle Hunter-Behrend, Erin Cullnan, Rebecca Higbee, Caleb Phillips, Sarah Williams, Philips Perera, Laleh Gharahbaghian

**Affiliations:** * Stanford University Medical Center, Department of Emergency Medicine, Palo Alto, California; † University of Colorado, Department of Computer Science, Boulder, Colorado

## Abstract

**Introduction:**

The focused assessment with sonography in trauma (FAST) exam is a critical diagnostic test for intraperitoneal free fluid (FF). Current teaching is that fluid accumulates first in Morison’s pouch. The goal of this study was to evaluate the “sub-quadrants” of traditional FAST views to determine the most sensitive areas for FF accumulation.

**Methods:**

We analyzed a retrospective cohort of all adult trauma patients who had a recorded FAST exam by emergency physicians at a Level I trauma center from January 2012 – June 2013. Ultrasound fellowship-trained faculty with three emergency medicine residents reviewed all FAST exams. We excluded studies if they were incomplete, of poor image quality, or with incorrect medical record information. Positive studies were assessed for FF localization, comparing the traditional abdominal views and on a sub-quadrant basis: right upper quadrant (RUQ)1 - hepato-diaphragmatic; RUQ2 - Morison’s pouch; RUQ3 - caudal liver edge and superior paracolic gutter; left upper quadrant (LUQ)1 - splenic-diaphragmatic; LUQ2 - spleno-renal; LUQ3 – around inferior pole of kidney; suprapubic area (SP)1 - bilateral to bladder; SP2 - posterior to bladder; SP3 – posterior to uterus (females). FAST results were confirmed by chart review of computed tomography results or operative findings.

**Results:**

Of the included 1,008 scans, 48 (4.8%) were positive. The RUQ was the most positive view with 32/48 (66.7%) positive. In the RUQ sub-quadrant analysis, the most positive view was the RUQ3 with 30/32 (93.8%) positive.

**Conclusion:**

The RUQ is most sensitive for FF assessment, with the superior paracolic gutter area around the caudal liver edge (RUQ3) being the most positive sub-quadrant within the RUQ.

## INTRODUCTION

The focused assessment with sonography in trauma (FAST) exam is a critical screening tool for intraperitoneal free fluid (FF) assessment from traumatic injury by evaluating the subxiphoid, right upper quadrant (RUQ), left upper quadrant (LUQ), and suprapubic (SP) areas.^1^–^3^ It is commonly taught that FF will first accumulate in the most dependent parts of the abdomen and pelvis in a supine trauma patient, specifically the RUQ and pelvis.^1^,^4^ The hepato-renal space (Morison’s pouch) has been concluded to be the primary area where FF is initially seen.^4^ Therefore, much of the current emphasis on the performance of the FAST exam has been placed on the RUQ Morison’s pouch view.^1^,^5^

**Figure 1:**
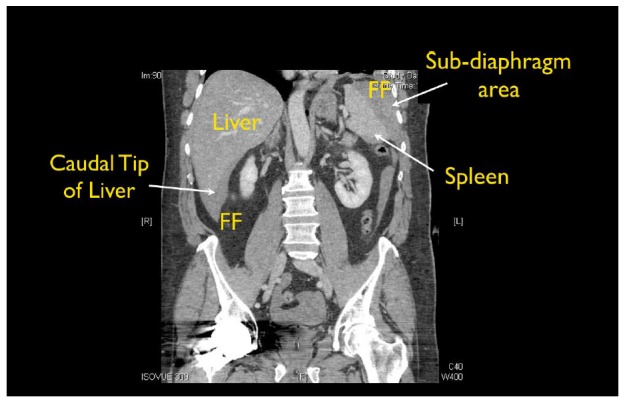
Computed tomography showing accumulation of free fluid (FF) in a traumatic supine patient.

Interestingly, few studies have specifically looked at where FF preferentially accumulates within each standard view of the FAST exam. In 1998, Rozycki et al. assessed the sensitivity of Morison’s pouch for the detection of FF, but did not analyze the sensitivity of other anatomic areas of the RUQ, nor the sensitivity of the other standard FAST views.^1^ In 1996, Lentz et al. examined abdominal ultrasound (US) exams to assess where fluid typically is seen within each quadrant, but the study was performed by US technicians and before the standardization of the FAST exam.^6^ Patient position is also important in adequate FF assessment. Several radiology studies using computed tomography (CT) and US scans have illustrated that FF layers to the most dependent areas in a supine patient (Figure 1), and best seen in the RUQ. 7,8

We determined the test characteristics of the subquadrants of the FAST exam compared to criterion reference of CT done immediately after the FAST was performed. Our goal was to investigate the traditional FAST views of the abdomen and pelvis, as well as perform a sub-quadrant analysis of the RUQ, LUQ and SP areas to better define FF localization in order to determine where to better focus the FAST exam in the trauma patient.

## METHODS

We analyzed a retrospective cohort of all adult trauma patients with recorded FAST exams by emergency medicine (EM) resident physicians of all levels of training at a Level 1 trauma center from January 2012 – June 2013. One US fellowship-trained faculty with three EM senior resident physicians reviewed all recorded FAST exams on supine adult trauma patients. Each FAST exam enrolled in the study had to include complete intraperitoneal views of sufficient quality to confidently assess all regions for FF by the reviewers. We excluded studies if all three intraperitoneal FAST views were not performed and/or recorded, image quality was extremely poor such that reviewers were unable to effectively assess the sub-quadrants, or accurate medical record information was not available for chart review of CT and operative findings. A study was positive if any amount of FF was noted in the peritoneum, including pelvis view of female patients. Positive studies were further evaluated to assess intraperitoneal FF location among the traditional abdominal and pelvic views of the FAST exam, and then further subdivided into the sub-quadrant areas. These areas included the originally described dependent areas of the abdomen: hepato-renal space, spleno-renal space and the pelvis as described by the first authors of the FAST exam 2 but also adjacent areas where FF has been noticed in clinical practice.

**Figure 2:**
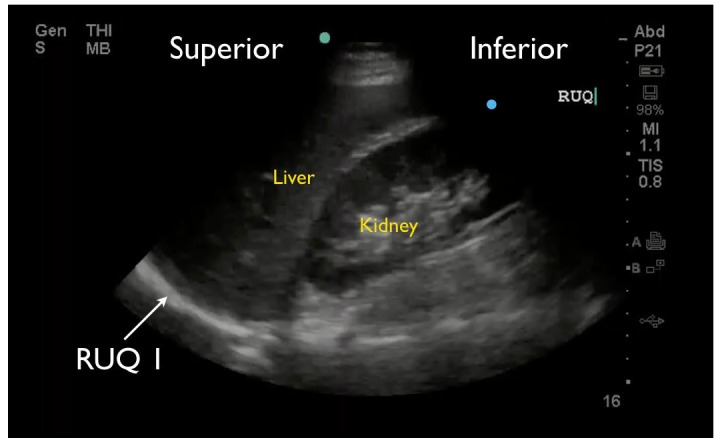
Right upper quadrant FAST view showing hepato-diaphragmatic space.

**Figure 3:**
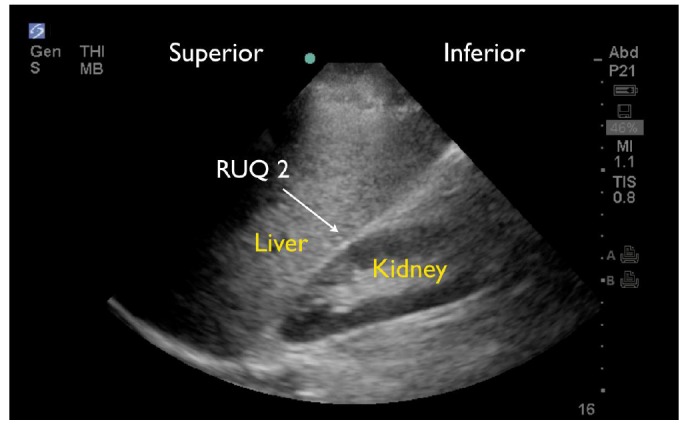
Normal right upper quadrant FAST view showing no free fluid in Morison’s pouch (RUQ2).

**Figure 4:**
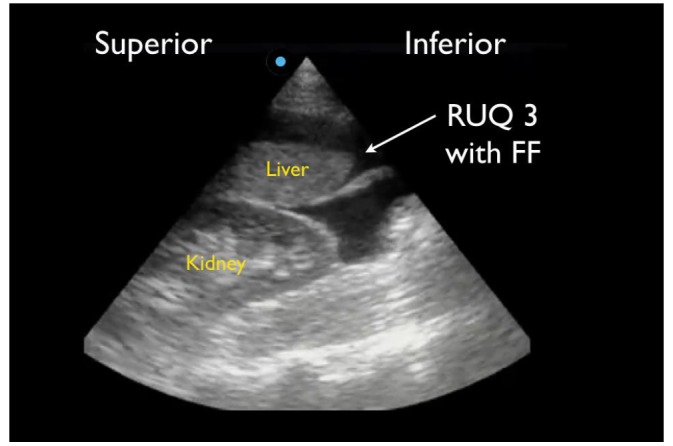
Positive right upper quadrant (RUQ) FAST view showing superior paracolic gutter around caudal liver edge (RUQ3), the most sensitive region for detecting free fluid (FF).

**Figure 5:**
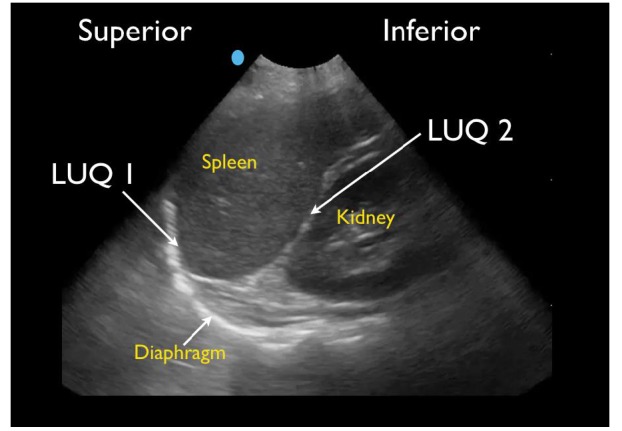
Normal left upper quadrant FAST view showing spleno-diaphragmatic space (LUQ1) and spleno-renal space (LUQ2).

**Figure 6:**
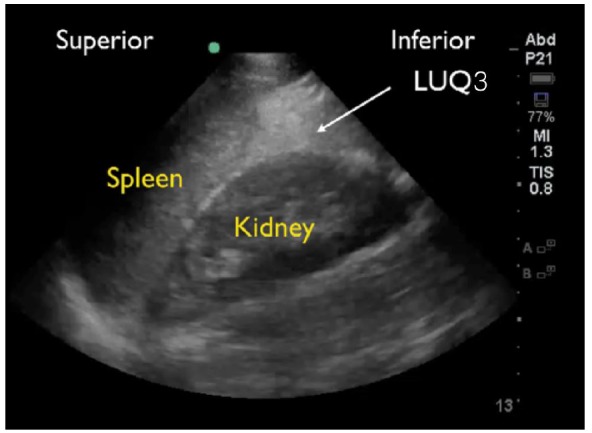
Normal left upper quadrant view of FAST showing left paracolic gutter (LUQ3).

**Figure 7:**
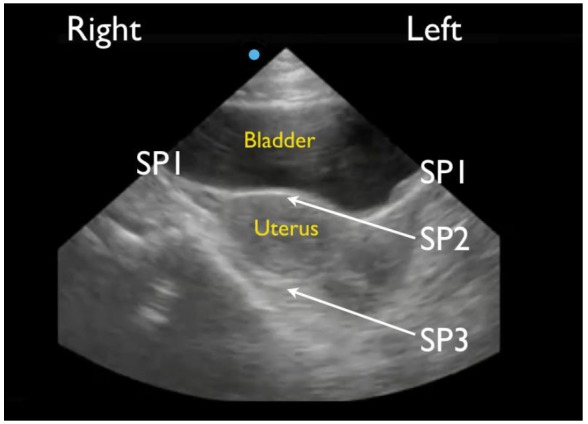
Normal short-axis suprapubic view of the FAST in a female showing lateral spaces to the bladder (SP1), space in between the bladder and uterus (SP2) and space posterior to the uterus (SP3).

**Figure 8:**
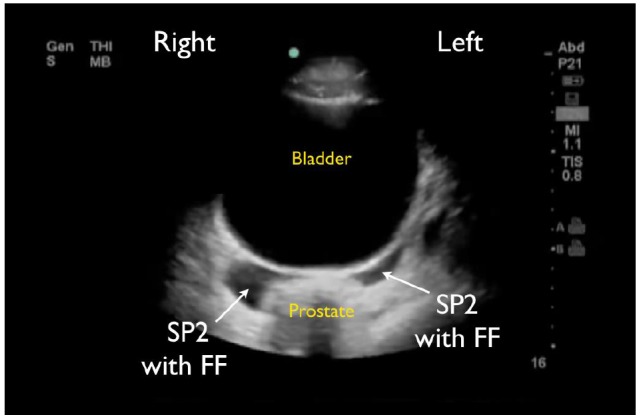
Suprapubic FAST view in a male patient showing free fluid (FF) posterior to bladder space but anterior to the prostate (SP2).

Sub-quadrants:

RUQ1 - hepato-diaphragmatic space: area between diaphragm and liver (Figure 2)
RUQ2 - hepato-renal space, or Morison’s pouch: area between liver and kidney (Figure 3)
RUQ3 – caudal edge of the liver, superior right paracolic gutter area (Figure 4)
LUQ1 - spleno-diaphragmatic space: area between spleen and diaphragm (Figure 5)
LUQ2 - spleno-renal space: area between spleen and kidney (Figure 5)
LUQ3 - inferior pole of the left kidney, or left paracolic gutter (Figure 6)
SP1 - lateral on either or both sides of bladder (Figure 7)
SP2 - posterior to bladder and anterior pelvic organs (Figure 8)
SP3 - posterior to uterus, or pelvic cul-de-sac, females only (Figure 7)

We reviewed medical records to confirm positive FAST results by noting the correlative findings on CT of the abdomen and pelvis. CTs were performed immediately after the trauma survey per ATLS guidelines, and read by board-certified radiologists. If a CT was not done, operative findings were compared. We plotted the percentage of positive sub-quadrants against the total number of positive studies evaluated with calculated percentages. All images reviewed were recorded using a SonoSite M-Turbo US machine using a phased array 5-1 MHz transducer.

We used Cohen kappa matrix and a pair-wise proportions test with Bonferroni correction for p values to evaluate for the correlation between quadrants and sub-quadrants. We assessed for statistically significant sensitivity of FF within sub-quadrants, and for predicting a positive quadrant.

The institutional review board approved the protocol, and appropriate protection of all medical health information was conducted.

## RESULTS

**Figure 9:**
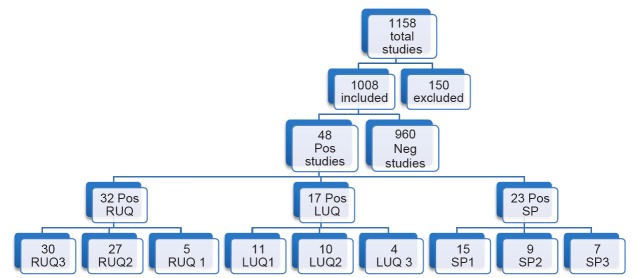
Flow chart of patient enrollment in retrospective study demonstrating caudal edge of the liver in the right upper quadrant view is the most sensitive area for free fluid on the FAST exam. *LUQ,* left upper quadrant; *LUQ1,* spleno-diaphragmatic space; *LUQ2,* spleno-renal space; *LUQ3*, inferior pole of the left kidney; *RUQ*, right upper quadrant; *RUQ1*, hepato-diaphragmatic space; *RUQ2*, hepato-renal space; *RUQ3,* caudal edge of the liver; *SP*, Supra-pubic; *SP1,* lateral on either or both sides of bladder; *SP2*, posterior to bladder and anterior pelvic organs; *SP3,* posterior to uterus, or pelvic cul-de-sac.

**Figure 10:**
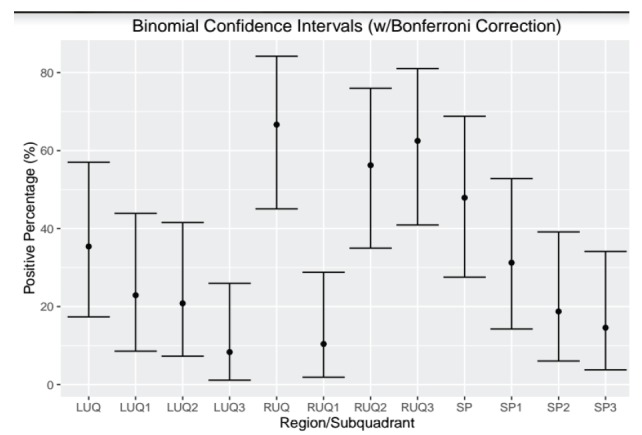
This plot gives the percentage of positive results from each sub-quadrant and quadrant with 95% confidence intervals around those estimates using the method of Clopper and Pearson with a Bonferroni correction to account for the multiple comparisons. As noted, the right upper quadrant (RUQ) is the most positive quadrant, and the caudal edge of the liver (RUQ3) is the most positive sub-quadrant among all reported FAST exams. *LUQ,* left upper quadrant; *LUQ1,* spleno-diaphragmatic space; *LUQ2,* spleno-renal space; *LUQ3*, inferior pole of the left kidney; *RUQ*, right upper quadrant; *RUQ1*, hepato-diaphragmatic space; *RUQ2*, hepato-renal space; *RUQ3,* caudal edge of the liver; *SP*, Supra-pubic; *SP1,* lateral on either or both sides of bladder; *SP2*, posterior to bladder and anterior pelvic organs; *SP3,* posterior to uterus, or pelvic cul-de-sac; *FAST*, focused assessment with sonography in trauma exams.

**Figure 11:**
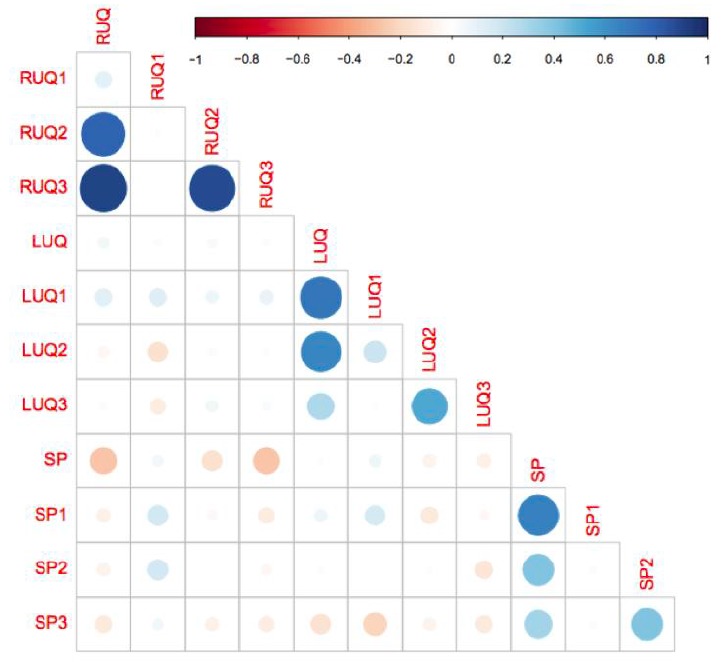
This matrix gives the correlation coefficient between pairs of quadrants and sub-quadrants using Cohen’s kappa. The kappa coefficient measures inter-rater agreement between qualitative (categorical items). Values of kappa range from −1 (indicating total disagreement) to 1 (indicating total agreement). In this plot, the size and color (redness/blueness) of the dots corresponds to the degree of positive or negative correlation. Hence, the small light dots have a correlation nearer zero, i.e., no discernable correlation. The darker larger more saturated dots have a correlation nearer 1 (blue) or −1 (red) meaning a stronger correlation. LUQ1 appears to be the most consistent with other quadrants, while SP3 is the most in disagreement. RUQ1 is sub-quadrant with the least in agreement with its containing quadrant (RUQ). *LUQ,* left upper quadrant; *LUQ1,* spleno-diaphragmatic space; *LUQ2,* spleno-renal space; *LUQ3*, inferior pole of the left kidney; *RUQ*, right upper quadrant; *RUQ1*, hepato-diaphragmatic space; *RUQ2*, hepato-renal space; *RUQ3,* caudal edge of the liver; *SP*, Supra-pubic; *SP1,* lateral on either or both sides of bladder; *SP2*, posterior to bladder and anterior pelvic organs; *SP3,* posterior to uterus, or pelvic cul-de-sac.

We reviewed a total of 1,158 FAST exams of adult (over 18 years of age) trauma patients over the study period. Of the 1,158 completed FAST exams, we excluded 150 (12.9%) exams due to incomplete saved exams (40%), poor image quality (35%) and incorrect medical record information (25%). The remaining 1,008 FAST scans were included for analysis, of which 48 (4.8%) were positive for hemoperitoneum (Figure 9). Among the positive studies, 39 (81%) of patients had a follow-up CT that confirmed the FAST findings, while 9 (19%) were taken emergently to the OR where hemoperitoneum was confirmed. There were no false positive FAST scans. In the traditional FAST views, 32/48 (66.7%) were positive for FF in the RUQ, 17/48 (35.4%) were positive in the LUQ, and 23/48 (47.9%) were positive in the SP region. Given that our study only focused on assessing for hemoperitoneum, the pericardial view of the FAST exam was not assessed. In sub-quadrant analysis of the RUQ, 30/32 (93.8%) were positive in RUQ3, 27/32 (84.4%) in RUQ2, and 5/32 (15.6%) in RUQ1. In the sub-quadrant analysis of the LUQ, 11/17 (64.7%) were positive in LUQ1, 10/17 (58.8%) in LUQ2, and 4/17 (23.5%) in LUQ3. In the sub-quadrant analysis of the SP view, there were 14 males and 9 females, of which 15/23 (64.7%) were positive in SP1, 9/23 (58.8%) in SP2 and 7/9 (77.7%) in SP3. The RUQ is the most sensitive region for FF assessment, followed by SP and LUQ. Within the RUQ, RUQ1 stands out as being the least sensitive with a substantial difference from the other RUQ sub-quadrants (Figure 10). Using Cohen kappa matrix (Figure 11), the correlation between quadrants and sub-quadrants can be shown. The RUQ is the most positive region of the FAST quadrants. The RUQ3 is the most sensitive indicator for a positive RUQ. LUQ1 is the most sensitive of the LUQ sub-quadrants and SP1 is the most sensitive of the SP sub-quadrants. Separate quadrants (i.e., RUQ, LUQ vs. SP) do not appear correlated in their positivity or negativity; for example, a positive RUQ does not necessarily mean a positive LUQ also.

**Figure 12:**
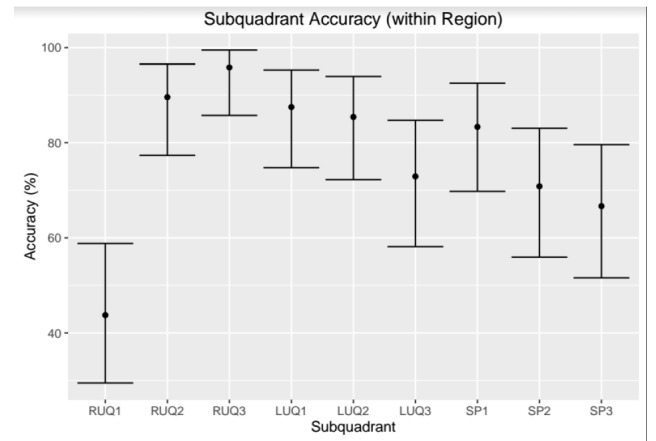
This plot gives the accuracy of each sub-quadrant in predicting the assessment from the corresponding full quadrant. Error bars describe the 95% confidence interval around the accuracy determined using the method of Clopper and Pearson. As depicted, the RUQ3 is the most accurate predictor of the RUQ. *LUQ,* left upper quadrant; *LUQ1,* spleno-diaphragmatic space; *LUQ2,* spleno-renal space; *LUQ3*, inferior pole of the left kidney; *RUQ*, right upper quadrant; *RUQ1*, hepato-diaphragmatic space; *RUQ2*, hepato-renal space; *RUQ3,* caudal edge of the liver; *SP*, Supra-pubic; *SP1,* lateral on either or both sides of bladder; *SP2*, posterior to bladder and anterior pelvic organs; *SP3,* posterior to uterus, or pelvic cul-de-sac.

Within each quadrant, the sub-quadrant accuracy is between 64% (SP3) and 94% (RUQ3). RUQ1 is an outlier with accuracy in its region of 43%. There were two cases where only the RUQ1 region was visualized as being the only positive view within the RUQ sub-quadrants. This is likely due to poor fanning and/or recording of images; the corresponding CT results confirmed FF through all sub-quadrants of the RUQ. RUQ1 and LUQ3 do not do better in their quadrants than random chance, while all other sub-quadrants predict their quadrant outcome with statistical significance (Figure 12).

## DISCUSSION

The accuracy of the FAST exam depends on multiple factors. It is important that the physician performing the FAST scan be skilled to correctly identify the various anatomical landmarks to assess for FF in the intraperitoneal, pleural and pericardial spaces. This study illustrates that the caudal liver edge and the superior aspect of the right paracolic gutter is the most sensitive indicator for FF in the intraperitoneal space, and not in Morison’s pouch as traditionally described. This is a critical finding and supports a change to the current teaching and performance of the FAST exam.

The trauma patient can arrive to an emergency department at any time period post-trauma, either ambulatory through the waiting room or supine by emergency medical services transport. Early scanning and patient positioning both provide potential obstacles to the ability to identify intraperitoneal FF. Fluid can accumulate over time in amounts needed to be visible on FAST scan, and in the region where FF is seen best: the RUQ in a supine patient.^9^ In a study evaluating FF location on supine patients by using CT imaging, Wojtowicz et al. noted that FF ascends and settles in the RUQ and pelvis. The FAST exam is often performed in the emergent trauma setting during or after the primary survey per ATLS protocol,^10^ where multiple evaluations and resuscitative measures are occurring simultaneously when a team-based approach is used. A higher-powered study assessing for the importance of serial FAST scans confirms that in supine patients, fluid accumulates over time, increasing one’s ability to detect hemoperitonuem.^11^

A recent study of blunt abdominal trauma patients showed the FAST scan as the best bedside diagnostic modality to identify intra-abdominal pathology.^11^ The FAST exam is ideal for detecting FF caused by intra-abdominal injury that results in shock and the need for emergent laparotomy.^3^,^12^ This validates the importance of the exam to be performed both rapidly, to facilitate the flow of trauma resuscitation, and thoroughly, to avoid inaccurate interpretation.

An experienced sonographer can detect just 600ml of intraperitoneal FF, and possibly even less with optimal pelvic views.^12^,^1^. To optimize the ability of locating small amounts of FF, it is important to obtain images from multiple intraperitoneal sites.^13^ As our study illustrates, FF may be seen in one quadrant but not others.

Patient positioning can affect the accuracy of the FAST scan. Various studies assessing supine vs. Trendelenburg positioning showed Trendelenburg positioning can allow detection of a lower amount of fluid (400 cc) as compared to the supine position (700cc).^15^,^16^ When patient fluid assessment is performed by US, the Trendelenburg and right decubitus positions improved visualization in the RUQ. This suggests that fluid shifts in the direction of gravity.^17^,^18^

Importantly, when assessing the intraperitoneal space using the traditional RUQ, LUQ and SP views, the physician must understand the most sensitive regions for visualizing FF accumulation in order to increase the sensitivity of the study. In supine patients, fluid will accumulate in the most dependent areas of the peritoneal cavity, which have been shown to be the RUQ and SP regions, leading to conventional teaching describing the RUQ, specifically Morison’s pouch, as the area where FF is first seen.^1^,^4^,^5^,^11^ We specifically designed our retrospective study to test the hypothesis that a methodical sub-quadrant analysis of the traditional FAST views may allow for improved detection of intraperitoneal FF on the FAST examination. The RUQ view is noted to be the most sensitive for intraperitoneal fluid in our study, confirming previous studies. The liver and kidney allow sound-wave penetration and prevent scatter, allowing for optimal images. This study illustrates that the caudal liver edge and the superior aspect of the right paracolic gutter and not Morison’s pouch is the most sensitive indicator for FF. While this difference between RUQ3 positivity and RUQ2 positivity was not statistically significant in our study, the RUQ3 was statistically the most sensitive indicator of a positive RUQ. These data support the premise that FF does in fact ascend and accumulate in the RUQ, as described by prior radiology CT studies,^7^ by first moving around the caudal liver edge (RUQ 3) before ascending into Morison’s pouch (RUQ2). This is important in the patient with early intraperitoneal bleeding who may only have FF in RUQ3. This study suggests placing less emphasis solely on imaging Morison’s pouch and more emphasis on a more comprehensive exam that includes the caudal liver edge.

The LUQ, although thought to have adequate windows due to the spleen and kidney, is less sensitive for the detection of FF in our study. The spleen is smaller than the liver and offers less of an acoustic window. Furthermore, the stomach intrudes in the image causing scatter artifact. The area between the diaphragm and the spleen, or LUQ1, was found to be the most sensitive area for the detection of FF out of the three LUQ sub-quadrants. This observation would agree with other studies that the LUQ is not a mirror image of the RUQ and must be examined differently. There were, however, five cases in our series where the LUQ was positive, but the RUQ was negative. Therefore, it must still be included in the FAST scan to increase the overall FAST accuracy.

While the pelvic region is the most dependent region in supine patients and can be a sensitive view for detecting FF, it can miss FF due to the difficulty in obtaining adequate images, especially when there is an empty bladder, bowel gas artifact scattering the image, or posterior acoustic enhancement distal to a full bladder. Furthermore, gender differences have been shown to affect where FF will accumulate. In males, intraperitoneal FF accumulates around the posterior wall of the bladder. In females, FF is seen posterior to the uterus, in the pouch of Douglas. This region can be sensitive in detecting very small amounts of fluids.^14^ However, small amounts of FF in young females of menstruating age can be normal in the absence of trauma, which further complicates traumatic FF assessment in this region.^19^ Our study found that lateral to the bladder (SP1) was the most sensitive SP region. However, our sample size by gender was small: 14 male and 9 female, limiting our analysis and conclusions.

## LIMITATIONS

Our study was a retrospective study with a small positive FAST cohort. This does not reflect a true measurement of the percentage of positive traumatic FAST scans at our institution, as there are scans not recorded due to time constraints in data entry and lack of reliable operator recording. Secondly, while all enrolled patients were evaluated while they were supine, the amount of time between their traumatic event and the FAST scan was not recorded, nor was the time until CT or operating room (OR) confirmation reported. In addition, patients are always taken to CT scanners and/or the OR after the initial FAST exam; this allows time for continued bleeding and new areas of FF that may have not been present at the time of the FAST scan. This difference will likely lead to a decrease in sensitivity of each sub-quadrant. Next, the same investigators reviewed all studies, which included one US fellowship-trained EM attending and three EM senior resident physicians. We did not perform inter-rater reliability testing although we did review the chart to confirm their results. Next, while our study assessed all traumatic patients, we did not correlate the specific injury type to the FAST findings. The study did not include the pericardial view, which is a normal component of the FAST exam. Finally, our small sample size of positive findings in the pelvis limited our ability to confidently discriminate between men and women. This view would have to be investigated according to gender in a larger sample size, as traumatic fluid accumulation differs between men and women based on the difference in pelvic organs.

## CONCLUSION

Compared to criterion references of CT and operative findings, we found that the sub-quadrants of the FAST scan most sensitive for FF visualization are RUQ3 (caudal tip of liver). RUQ3 is always positive when Morison’s pouch (RUQ 2) was positive for FF, but fluid may be seen here without being seen in Morison’s pouch. This represents a change from the prior emphasis placed on Morison’s pouch during performance and teaching of the FAST exam.
